# Supplementary material for: How Depressing Is Inbreeding? A Meta-Analysis of 30 Years of Research on the Effects of Inbreeding in Livestock
Source: Genes (Basel). 2021 Jun 18;12(6):926. doi: 10.3390/genes12060926 (PMC8234567; doi:10.3390/genes12060926)
Supplement: Supplementary file 1 [file genes-12-00926-s001.zip › Table_S3.pdf]

Table S3 Pairwise comparisons of estimated marginal means (EMMs) of b_m between trait groups*. The EMM for each trait group is shown (on diagonal), as well as the difference between two EMMs (below diagonal) and Tukey's adjusted P-value (above diagonal). Significant differences (P < 0.05) are shown in bold.

	REP/SUR	WEI/GRO	CONF	PROD	HEA	OTH
REP/SUR	*-0.302*	0.673	**0.010**	1.000	1.000	**0.001**
WEI/GRO	-0.075	*-0.227*	0.742	0.724	0.999	**0.013**
CONF	**-0.160**	-0.085	*-0.142*	**0.029**	0.821	0.103
PROD	0.006	0.080	**0.166**	*-0.308*	0.999	**0.001**
HEA	-0.034	0.041	0.126	-0.040	*-0.268*	0.053
OTH	**-0.431**	**-0.356**	-0.271	**-0.436**	-0.397	*0.129*

*REP/SUR: reproduction/survival, WEI/GRO: weight/growth, CONF: conformation, PROD: production, HEA: health, OTH: other
