# Supplementary material for: How Depressing Is Inbreeding? A Meta-Analysis of 30 Years of Research on the Effects of Inbreeding in Livestock
Source: Genes (Basel). 2021 Jun 18;12(6):926. doi: 10.3390/genes12060926 (PMC8234567; doi:10.3390/genes12060926)
Supplement: Supplementary file 1 [file genes-12-00926-s001.zip › Table_S4.pdf]

Table S4 Pairwise comparisons of estimated marginal means (EMMs) of b_s between trait groups*. The EMM for each trait group is shown (on diagonal), as well as the difference between two EMMs (below diagonal) and Tukey's adjusted P-value (above diagonal). Significant differences (P < 0.05) are shown in bold.

	REP/SUR	WEI/GRO	CONF	PROD	HEA	OTH
REP/SUR	*-0.410*	0.311	0.999	0.735	0.919	0.117
WEI/GRO	0.661	*-1.071*	0.535	0.943	1.000	**0.004**
CONF	0.077	-0.584	*-0.487*	0.908	0.957	0.064
PROD	0.343	-0.318	0.266	*-0.753*	1.000	**0.021**
HEA	0.481	-0.180	0.404	0.138	*-0.891*	0.086
OTH	-1.236	**-1.897**	-1.313	**-1.579**	-1.717	*0.826*

*REP/SUR: reproduction/survival, WEI/GRO: weight/growth, CONF: conformation, PROD: production, HEA: health, OTH: other
