# Supplementary material for: How Depressing Is Inbreeding? A Meta-Analysis of 30 Years of Research on the Effects of Inbreeding in Livestock
Source: Genes (Basel). 2021 Jun 18;12(6):926. doi: 10.3390/genes12060926 (PMC8234567; doi:10.3390/genes12060926)
Supplement: Supplementary file 1 [file genes-12-00926-s001.zip › Table_S5.pdf]

Table S5 Estimated marginal means of inbreeding depression estimates per trait, with standard errors (SEs). Inbreeding depression estimates are expressed as percentage of the trait mean (b_m) or as percentage of the trait standard deviation (b_s). The number of estimates (N) and the P-value for testing the mean against zero are also shown. Only traits with at least 10 records were included in the analysis.

		b_m			b_s		
**Trait group***	**Trait**	**N**	**Estimate (SE)**	**P-value**	**N**	**Estimate (SE)**	**P-value**
REP/SUR	Fertility interval	154	-0.271 (0.055)	<0.001	129	-0.678 (0.255)	0.008
REP/SUR	Fertility success	93	-0.413 (0.072)	<0.001	62	-0.554 (0.375)	0.139
REP/SUR	Fertility success (offspring)	19	-0.465 (0.154)	0.003	-	-	-
REP/SUR	Fertility success (sire)	10	-0.293 (0.211)	0.165	-	-	-
REP/SUR	Gestation length	28	0.000 (0.108)	1.000	24	0.057 (0.459)	0.902
REP/SUR	Calving ease	26	0.088 (0.124)	0.481	14	1.007 (0.595)	0.091
REP/SUR	Litter size	26	-0.216 (0.123)	0.020	17	-0.596 (0.711)	0.401
REP/SUR	Litter size (maternal)	41	-0.186 (0.104)	0.075	20	0.848 (0.754)	0.261
REP/SUR	Litter size at later stage	11	-0.512 (0.178)	0.004	-	-	-
REP/SUR	Litter size at later stage (maternal)	15	-0.576 (0.158)	<0.001	-	-	-
REP/SUR	Adult survival	11	-0.197 (0.211)	0.349	-	-	-
REP/SUR	Offspring survival	51	-0.256 (0.093)	0.006	-	-	-
REP/SUR	Offspring survival (maternal)	52	-0.153 (0.088)	0.082	14	1.522 (0.588)	0.010
REP/SUR	Longevity	21	-0.938 (0.125)	<0.001	23	-1.341 (0.484)	0.006
WEI/GRO	Birth weight	61	-0.173 (0.079)	0.029	32	-1.024 (0.462)	0.027
WEI/GRO	Birth weight (maternal)	34	-0.034 (0.101)	0.739	15	-0.627 (0.603)	0.299
WEI/GRO	Weight	191	-0.230 (0.052)	<0.001	105	-1.179 (0.292)	<0.001
WEI/GRO	Weight (maternal)	61	-0.170 (0.081)	0.035	36	-0.740 (0.420)	0.078
WEI/GRO	Growth	45	-0.196 (0.091)	0.032	27	-0.133 (0.460)	0.773
WEI/GRO	Growth (maternal)	17	0.170 (0.143)	0.234	-	-	-
CONF	Body dimensions	226	-0.197 (0.062)	0.004	212	-0.737 (0.269)	0.006
CONF	Body dimensions (maternal)	20	-0.061 (0.143)	0.669	12	0.356 (0.638)	0.577
CONF	Bone quality	19	-0.246 (0.134)	0.067	18	-0.315 (0.542)	0.561
CONF	Conformation dairy	84	-0.117 (0.075)	0.121	90	-0.278 (0.315)	0.378
CONF	Conformation other	52	-0.112 (0.088)	0.203	52	-0.536 (0.360)	0.137
CONF	Scrotal circumference	17	-0.172 (0.140)	0.217	11	-1.200 (0.669)	0.073
PROD	Milk yield	69	-0.547 (0.081)	<0.001	52	-1.587 (0.377)	<0.001
PROD	Fat yield	39	-0.507 (0.100)	<0.001	32	-1.903 (0.435)	<0.001
PROD	Protein yield	37	-0.452 (0.103)	<0.001	30	-1.938 (0.446)	<0.001
PROD	Fat%	21	-0.099 (0.130)	0.448	18	0.239 (0.553)	0.665
PROD	Protein%	20	-0.070 (0.134)	0.602	17	1.057 (0.570)	0.064
PROD	Milk other	12	-0.155 (0.163)	0.340	-	-	-
PROD	Egg number	17	-0.447 (0.192)	0.020	10	-1.889 (1.593)	0.236
PROD	Egg weight	16	-0.290 (0.167)	0.083	-	-	-
PROD	Carcass/meat quality	21	-0.054 (0.128)	0.675	13	0.384 (0.636)	0.546
PROD	Fleece production	10	-0.251 (0.211)	0.235	-	-	-
PROD	Litter weight (maternal)	11	-0.203 (0.184)	0.271	-	-	-
PROD	Litter weight at weaning	10	-0.878 (0.183)	<0.001	-	-	-
PROD	Litter weight at weaning (maternal)	11	0.144 (0.172)	0.403	-	-	-
PROD	Production other	12	-0.803 (0.422)	0.422	-	-	-
HEA	SCS	22	-0.609 (0.129)	<0.001	21	-0.501 (0.522)	0.337
OTH	Behavior	14	-0.005 (0.150)	0.972	13	0.412 (0.614)	0.503
OTH	Horse competition performance	-	-	-	10	-0.602 (0.848)	0.478

*REP/SUR: reproduction/survival, WEI/GRO: weight/growth, CONF: conformation, PROD: production, HEA: health, OTH: other
